# A step forward in antibiotic use and resistance monitoring: a quarterly surveillance system pilot in 11 European Union/European Economic Area countries, September 2017 to May 2020

**DOI:** 10.2807/1560-7917.ES.2022.27.46.2200082

**Published:** 2022-11-17

**Authors:** Germán Peñalva, Paloma Crespo-Robledo, Mari Molvik, Antonio López-Navas, Oliver Kacelnik, José Miguel Cisneros, EU-JAMRAI WP7.4.1 group, EU-JAMRAI WP7.4.1 group, Vera Buhmann, Anton Hlava, Blaženka Hunjak, Pero Ivanko, Anamarija Škoda, Helena Žemličková, Barbora Mackova, Sissel Skovgaard, Ute Wolff Sönksen, Arina Zanuzdana, Tim Eckmanns, Alkiviadis Vatopoulos, Michalis Polemis, Domenico Martinelli, Francesca Fortunato, Asta Dambrauskiene, Rolanda Valinteliene, Anna Olczak-Pieńkowska, Dorota Żabicka, Maria Rodrigues, Nuno Pereira, Isabel Neves, José Antonio Lepe, Raquel Valencia, María Victoria Gil-Navarro, Antonio Oliver-Palomo, Leonor del Mar Periañez-Parraga, Estrella Rojo-Molinero, Glòria Oliva, Marta Massanes, José Manuel Izquierdo-Palomares, Ainhoa Aranguren, Víctor José Rausell-Rausell, María del Pilar López-Acuña, Alberto Gil-Setas, María Eugenia Portillo

**Affiliations:** 1 Department of Infectious Diseases, Microbiology and Preventive Medicine, Institute of Biomedicine of Seville (IBiS), University Hospital Virgen del Rocío, CSIC, University of Seville, Seville, Spain; 2 Spanish Agency of Medicines and Medical Devices (AEMPS), National Action Plan against Antibiotic Resistance (PRAN), Madrid, Spain; 3 Department for Infection Prevention and Preparedness, Norwegian Institute of Public Health (FHI), Oslo, Norway; 4 Additional members of the EU-JAMRAI WP7.4.1 group are listed under Collaborators and at the end of the article

**Keywords:** Surveillance, EU-JAMRAI, antimicrobial resistance, antibiotic use, Europe

## Abstract

**Background:**

Surveillance of antimicrobial resistance (AMR) and antimicrobial use (AMU) in Europe is currently annual.

**Aim:**

To study the feasibility and scalability of a quarterly AMR/AMU surveillance system in the European Union/European Economic Area (EU/EEA).

**Methods:**

We conducted a longitudinal study within the scope of the EU-JAMRAI project. Seventeen partners from 11 EU/EEA countries prospectively collected 41 AMU and AMR indicators quarterly from September 2017 to May 2020 for the hospital sector (HS) and primary care (PC). Descriptive statistics and coefficients of variation (CV) analysis were performed.

**Results:**

Data from 8 million hospital stays and 45 million inhabitants per quarter were collected at national (n = 4), regional (n = 6) and local (n = 7) levels. Of all partners, five were able to provide data within 3 months after each preceding quarter, and eight within 3–6 months. A high variability in AMU was found between partners. Colistin was the antibiotic that showed the highest CV in HS (1.40; p < 0.0001). Extended-spectrum beta-lactamase-producing *Escherichia coli* presented the highest incidence in HS (0.568 ± 0.045 cases/1,000 bed-days per quarter), whereas ciprofloxacin-resistant *E. coli* showed the highest incidence in PC (0.448 ± 0.027 cases/1,000 inhabitants per quarter). Barriers and needs for implementation were identified.

**Conclusion:**

This pilot study could be a first step towards the development of a quarterly surveillance system for AMU and AMR in both HS and PC in the EU/EEA. However, committed institutional support, dedicated human resources, coordination of data sources, homogeneous indicators and modern integrated IT systems are needed first to implement a sustainable quarterly surveillance system.

**Key public health message**

**What did you want to address in this study?**

In the European Union/ European Economic Area, surveillance data on antimicrobial resistance (AMR) and antimicrobial use (AMU) are collected on an annual basis. We wanted to address the feasibility of developing a quarterly surveillance system for AMU and AMR in both hospitals and primary care.

**What have we learnt from this study?**

We identified that 13 of the 17 partners in the pilot project could submit data within 6 months. However, in order to increase the frequency at which AMU and AMR data are provided, committed institutional support, dedicated human resources, and coordination of microbiological and antimicrobial use data sources – which implies common indicators and modern integrated IT systems – are needed.

**What are the implications of your findings for public health?**

More frequent AMU and AMR surveillance reporting appears feasible in the EU/EEA to timely inform interventions at local, regional and national levels. However, there are some caveats that should be addressed before quarterly surveillance can be implemented at the European level.

## Introduction

The emergence and rapid global spread of antimicrobial resistance (AMR) is a major threat to patients, healthcare systems and the economy [1]. The overuse and misuse of antibiotics are major driving forces towards AMR [2,3]. Patients who are infected with antibiotic-resistant bacteria are more likely to develop complications and up to three times more likely to die from the infection [4]. In 2019, 1.27 million deaths were directly attributable to bacterial AMR worldwide [5]. In the European Union/European Economic Area (EU/EEA) alone, over 33,000 people die every year due to infections caused by antibiotic-resistant bacteria [6] and, if no action is taken, the global burden of deaths could dramatically increase [7]. The economic impact of AMR in the EU is estimated as 1.5 billion EUR per year in healthcare costs and productivity losses [8].

One of the strategic objectives to tackle AMR stated in the 2015 WHO Global Action Plan on Antimicrobial Resistance is to strengthen the knowledge and evidence base through surveillance and research [9]. In 2017, the European Commission communicated that better coordination and surveillance were considered as a part of the key objective of making the EU a best practice region against AMR [10]. In 2019, the Council of the EU highlighted the need to strengthen and widen the scope of surveillance of AMR and healthcare-associated infections (HCAI), as well as the consumption of antimicrobials, both in the human and the animal health sectors, fostering actions under a One Health approach [11,12].

Surveillance of AMR and antimicrobial use (AMU) is paramount in monitoring progress of AMR national action plans (NAPs) as well as the effectiveness of specific policies [13]. Currently, the EU has well-established national and international surveillance systems for AMR and AMU. The European Centre for Disease Prevention and Control (ECDC) manages and coordinates the European Antimicrobial Resistance Surveillance Network (EARS-Net) and the European Surveillance of Antimicrobial Consumption Network (ESAC-Net), monitoring both AMR and AMU surveillance data from the European Union/European Economic Area (EU/EEA) on a yearly basis [14,15]. However, considering that AMR is associated with the use of antimicrobials over time, surveillance data should be available at more frequent time intervals to inform health actions at the local, regional and national level, and to assess the effectiveness of interventions [1,9]. This is also vital for effective antimicrobial stewardship programmes [16], where sustained periodical monitoring and feedback have proved to attain favourable long-term prescribing behavioural change [17,18].

In order to reduce the current time gap between AMR and AMU data collection and assessment, a quarterly surveillance system in human health has been piloted during a 2.5-year period within the scope of the EU-JAMRAI, the European Union Joint Action on Antimicrobial Resistance and Healthcare-Associated Infections (https://eu-jamrai.eu). The aim of this study is to describe the methodology, present the outcomes of the piloting phase and assess the feasibility and scalability of a quarterly surveillance system in the EU.

## Methods

### Setting

The EU-JAMRAI was run from September 2017 to February 2021 with the objective of fostering synergies among EU/EEA countries by developing and implementing effective One Health policies to fight the rising threat of AMR and to reduce HCAI. Its Work Package 7, ‘Appropriate use of antimicrobials in healthcare’, included a specific task to ‘Develop and test near real-time surveillance of antimicrobials and multidrug-resistant bacteria in human health’. On the basis of the successful experience of an Institutional Programme for the prevention and control of Healthcare-Associated Infections and Appropriate Use of Antimicrobials in Andalusia, Spain (PIRASOA) [18-20], we performed a pilot study as a part of the joint action aiming to gather data on AMU and AMR on a quarterly basis in the EU/EEA.

### Study design and period

We conducted a longitudinal descriptive study. Data were prospectively collected from September 2017 to May 2020, spanning 11 quarters. Piloting phase indicators are described in the Supplementary material on page 9. Seventeen partners from 11 European countries participated in the study (see Supplementary Table S1 for a list of the institutions). The pilot study was coordinated by two co-leading institutions: the Spanish Agency of Medicines and Medical Devices and the Andalusian Health Service, with the support of the Norwegian Institute of Public Health. Start-up guidelines, proposed indicators, and data collection forms were presented to all partners. The AMU and AMR indicators were discussed among the interested institutions and a final list was agreed. A teleconference with ECDC, a key stakeholder, was held to discuss possible overlaps and synergies. A secure file transfer web platform to upload the quarterly data was created. The mandatory quarterly surveillance period spanned from the first quarter of 2018 to the fourth quarter of 2019. In addition, any institution that wished to extend their data submission to include the fourth quarter of 2017 and/or the first and second quarters of 2020 was allowed do so.

### Surveillance scope and frequency

The scope of surveillance covered local, regional and/or national levels and was divided up into two sublevels: hospital sector (HS) and primary care (PC). Data were meant to be collected locally and then provided in an aggregated form per region or country. Eight participants engaged to implement the pilot study in HS and PC, other seven only in HS, and other two only in PC (see Supplementary Table S2 for an overview of healthcare scope, geographical scope and surveillance coverage for each institution). Data from individual healthcare centres were considered as the starting point of the surveillance system for those partners that were not able to obtain aggregated indicators initially, while pursuing the objective of increasing the scope over the lifetime of the project.

The frequency of the surveillance was quarterly data updates and feedback. Data managers had a 3-month period after the end of each quarter to gather and report data.

### Indicators under surveillance

Forty-one indicators were selected: 19 AMU indicators for HS; 10 AMU indicators for PC; seven AMR indicators for HS; and five AMR indicators for PC. Data were entered by each participating region/country in templates with two figures per indicator: a numerator and a denominator (described below; see Supplementary Figures S1 and S2 for data collection form for HS and PC, respectively). Calculations were carried out by the software to minimise human error. Data collected at healthcare centres were merged and presented aggregated for HS and/or PC by each partner. The accuracy of data was under each national coordinator’s responsibility. Before inclusion in the database, quarterly AMU and AMR data submitted by each participant were validated within 1 month by a member of the coordination team in terms of type, range, format and consistency to inform the provider and correct them, if needed. Data were made available to the partners on a secured web platform immediately after validation.

#### Indicators for antibiotic use

AMU was assessed as defined daily doses (DDD) of prescribed antimicrobials per 1,000 occupied bed days (OBD or stays) per quarter in HS, and DDD per 1,000 inhabitants per quarter in PC settings. DDDs were calculated accordingly to the Anatomical Therapeutical Chemical Classification (ATC) methodology index. Data for the quarters of 2017 and 2018 were reported following the ATC-WHO-2018 calculations. Data for the quarters of 2019 and 2020 were reported following the new ATC-WHO-2019 calculations [21].

Overall, AMU in HS was calculated as total DDD of antibiotics for systemic use (ATC group J01) per quarter x 1,000 divided by the number of total OBD during the quarter. The key antibiotics for HS under surveillance were: piperacillin-tazobactam (J01CR05); amoxicillin/clavulanic acid (J01CR02); carbapenems (J01DH) (ertapenem + imipenem + meropenem + doripenem); third and fourth generation cephalosporins (J01DD and J01DE) (ceftriaxone + cefotaxime + ceftazidime + cefepime); fluoroquinolones (J01MA) (ciprofloxacin + levofloxacin + moxifloxacin); vancomycin (J01XA01); and colistin (J01XB01). Services that do not generate hospital stays (i.e. emergency, observation, day hospital, dialysis, etc.) were excluded.

Overall, AMU in PC was calculated as total DDD of antibiotics for systemic use (J01) per quarter x 1,000 divided by the number of inhabitants in the healthcare area during the quarter. The key antibiotics for PC under surveillance were: amoxicillin/clavulanic acid (J01CR02); fluoroquinolones (J01MA) (ciprofloxacin + levofloxacin + moxifloxacin); and macrolides (J01FA) (erythromycin + clarithromycin + azithromycin).

#### Indicators for antimicrobial resistance

AMR was assessed as incidence density of resistant bacteria, calculated as the number of isolates in all clinical samples per patient divided by 1,000 OBD per quarter in HS, and as the number of isolates in all clinical samples per patient divided by 1,000 inhabitants per quarter in PC. Duplicated bacterial strains in the same patient were not included in the surveillance, as well as those samples from environment and colonisation screening, as these outcomes would depend on the surveillance intensity of each health centre. For the determination of antimicrobial susceptibility testing, laboratories could use either automated methods or disk diffusion tests. The use of European Committee on Antimicrobial Susceptibility Testing (EUCAST) and EUCAST-related guidelines for the detection of resistance mechanisms was recommended [22,23].

The AMR indicators under surveillance in HS were the incidence density of carbapenemase-producing Enterobacterales (CPE), extended-spectrum beta-lactamase-producing (ESBL) *Escherichia coli*, ESBL *Klebsiella pneumoniae*, carbapenem-resistant (CR) *Acinetobacter baumannii*, CR-*Pseudomonas aeruginosa*, meticillin-resistant *Staphylococcus aureus* (MRSA) and vancomycin-resistant enterococci –*Enterococcus faecalis* and *Enterococcus faecium –* (VRE).

The AMR indicators under surveillance in PC were the incidence density of CPE, ciprofloxacin-resistant *E. coli*, ESBL *E. coli*, ESBL *K. pneumoniae* and MRSA.

### Statistical analysis

Descriptive statistics of the variables were presented as means and standard deviations (SD), ranges and coefficients of variation (CV). To analyse the statistical significance of the dispersion of each indicator among the participant institutions, we used the Feltz and Miller’s asymptotic test for the equality of CVs from *k* populations [24] by using the R [25] package ‘cvequality’ (Version 0.1.3) [26] to test for differences among the variables’ CVs during 2018 and 2019. Pooled analyses for AMR were calculated by averaging the quarterly sum of isolates of all partners x 1,000 divided by the sum of hospital OBD, for the eight quarters of the mandatory period 2018Q1–2019Q4.

## Results

Seventeen institutions from 11 European countries participated in the task (see Supplementary Table S1 for a list of the institutions). Fifteen partners provided HS data, whereas 10 provided PC data. The surveillance geographical scope ranged from 4/17 with national level data, 6/17 with regional level data and 7/17 with local data (1–3 hospitals or community healthcare areas). On average, the surveillance system collected data from nearly 8 million hospital stays in HS, and from 45 million inhabitants in PC per quarter (see Supplementary Table S2 for healthcare scope, geographical scope and surveillance coverage by each institution).

### Data submission time lag

The average time delay between the end of a quarter and submission of the corresponding data to the website ranged from < 3 months (5/17 participant institutions), 3–6 months (8/17), and > 6 months (4/17).

### Quarterly participation

#### Hospital sector quarterly surveillance

Eleven out of the 15 partners engaged in the HS surveillance provided data for both AMU and AMR. The remaining four provided only AMU data. Eleven partners provided data for all the quarters of 2018 and 2019; one of them could not provide hospital OBD for the denominator but only inhabitants and was excluded from the statistical analysis; six partners continued providing data during 2020. One institution provided data only for 2018. A second institution stopped data submission after the 2nd quarter of 2019 (see Supplementary Table S3 for an overview of quarterly surveillance data reporting in the HS). The indicators for HS provided per participating institution are shown in Supplementary Table S4.

#### Primary care quarterly surveillance

Seven of the 10 partners engaged in the PC surveillance provided data for both AMU and AMR. The remaining three provided only AMU data. All partners engaged in the PC surveillance provided quarterly data for 2018 and 2019; five of them continued providing data during 2020 (see Supplementary Table S5 for an overview of quarterly surveillance data reporting in PC). The indicators under surveillance for PC per participating institution are shown in Supplementary Table S6.

### Antibiotic use in the hospital sector

Table 1 shows the descriptive statistics of the quarterly use of antibiotics in HS. For the overall 2018–19 surveillance period, a high level of variability in AMU was found between partners, with CVs of 0.62 for amoxicillin/clavulanic acid, 0.59 for piperacillin/tazobactam, 0.53 for carbapenems, 0.61 for fluoroquinolones, 0.57 for third and fourth generation cephalosporins, 0.56 for vancomycin and 1.40 for colistin, which was the antibiotic with the highest rate of variability between participant institutions (p < 0.00001).

**Table 1:** Antibiotic use in the hospital sector in defined daily doses per 1,000 occupied bed-days per quarter, eight European countries, September 2017–May 2020 (n = 14 institutions)

Antibiotic	Quarters										
	2017	2018				2019				2020	
	Q4	Q1	Q2	Q3	Q4	Q1	Q2	Q3	Q4	Q1	Q2
Total antibiotics for systemic use (J01)											
N	6	14	14	14	14	13	13	11	10	5	3
Mean	767.9	721.4	693.9	689.5	719.3	684.6	631.0	685.9	682.5	689.5	848.9
SD	284.2	289.6	258.4	262.2	273.5	245.5	195.5	211.9	227.3	306.1	145.8
Min.	506.0	303.3	323.0	288.4	310.4	251.7	252.4	271.8	322.6	249.1	739.7
Max.	1,152.3	1,329.3	1,107.8	1,126.2	1,209.4	1,102.0	1,011.8	1,029.0	1,103.9	1,097.8	1,014.5
CV	0.37	0.40	0.37	0.38	0.38	0.36	0.31	0.31	0.33	0.44	0.17
Amoxicillin/clavulanic acid (J01CR02)											
N	6	13	13	13	13	12	12	10	9	4	3
Mean	98.6	113.4	107.0	99.5	106.5	102.0	93.8	96.6	111.6	133.4	132.8
SD	80.3	74.4	75.1	67.1	72.3	63.1	55.3	57.6	53.4	50.0	2.6
Min.	1.3	2.1	2.2	1.0	0.9	0.4	0.6	0.5	1.5	74.6	130.0
Max.	217.6	208.8	227.0	205.5	213.0	201.7	165.4	167.0	167.3	182.4	135.1
CV	0.81	0.66	0.70	0.67	0.68	0.62	0.59	0.60	0.48	0.37	0.02
Piperacillin/tazobactam (J01CR05)											
N	6	13	13	13	13	12	12	10	9	4	3
Mean	39.3	51.2	47.3	47.4	50.0	54.1	50.3	68.4	61.5	64.5	52.5
SD	30.8	36.2	27.7	31.9	31.0	33.8	33.5	28.8	32.1	21.4	32.4
Min.	8.8	7.4	11.0	7.5	9.2	11.7	11.4	20.8	19.8	48.7	16.9
Max.	96.0	124.0	101.9	111.0	115.2	120.5	110.5	117.8	126.8	96.1	80.2
CV	0.78	0.71	0.59	0.67	0.62	0.62	0.67	0.42	0.52	0.33	0.62
Carbapenems (J01DH)											
N	6	13	13	13	13	12	12	10	9	4	3
Mean	40.5	52.5	50.6	49.6	58.0	38.6	39.4	45.9	41.0	45.2	56.5
SD	23.9	29.7	26.4	26.4	37.6	16.7	15.5	18.2	15.7	9.8	1.5
Min.	18.7	16.4	15.3	17.3	21.5	16.6	20.9	19.8	16.2	34.1	54.9
Max.	81.9	106.0	100.0	107.8	158.7	73.6	71.8	82.4	69.0	54.4	57.8
CV	0.59	0.57	0.52	0.53	0.65	0.43	0.39	0.40	0.38	0.22	0.03
Fluoroquinolones (J01MA)											
N	6	14	14	14	14	13	13	11	10	5	3
Mean	94.4	93.8	77.8	76.2	74.5	77.2	63.1	64.1	67.1	95.5	93.9
SD	59.3	64.2	45.8	41.9	42.7	53.0	40.3	34.4	39.7	54.2	10.9
Min.	10.0	8.7	11.9	16.1	11.1	5.5	6.9	12.1	12.6	4.1	85.9
Max.	184.3	217.8	164.6	159.5	155.7	178.9	132.0	123.0	131.4	147.9	106.3
CV	0.63	0.68	0.59	0.55	0.57	0.69	0.64	0.54	0.59	0.57	0.12
Third and fourth generation cephalosporins (J01DD and J01DE)											
N	6	13	13	13	13	12	12	10	9	4	3
Mean	60.2	66.8	62.7	63.2	67.1	66.5	64.9	64.7	64.2	113.0	124.8
SD	46.5	42.6	36.2	37.0	38.6	41.5	34.9	36.1	41.0	35.6	49.4
Min.	14.4	12.0	12.2	13.3	12.9	12.6	12.1	13.7	10.6	73.9	67.7
Max.	130.4	156.6	128.5	133.0	132.5	146.3	122.5	127.7	139.2	159.6	154.5
CV	0.77	0.64	0.58	0.59	0.58	0.62	0.54	0.56	0.64	0.32	0.40
Vancomycin (J01XA01)											
N	6	13	13	13	13	12	12	10	9	4	3
Mean	15.3	13.7	13.9	13.3	15.0	14.5	14.8	15.3	15.1	16.8	16.3
SD	3.4	8.3	7.3	6.9	9.5	7.8	9.4	8.6	8.5	11.8	3.7
Min.	10.2	1.5	1.8	0.4	1.2	0.6	1.2	2.0	0.8	6.4	12.6
Max.	20.2	29.0	30.0	27.1	36.4	31.8	40.4	33.5	32.4	33.8	20.0
CV	0.22	0.60	0.52	0.52	0.63	0.54	0.64	0.56	0.57	0.70	0.23
Colistin (J01XB01)											
N	6	13	13	13	13	12	11	10	9	4	3
Mean	14.1	13.4	13.6	13.9	13.6	9.9	9.9	13.0	10.9	20.0	29.0
SD	25.5	17.7	19.9	18.1	17.9	15.5	13.2	20.1	20.2	37.0	44.6
Min.	0.5	0.2	0.2	0.1	0.1	0.7	0.2	0.8	0.3	0.5	1.6
Max.	65.9	58.2	64.2	65.2	62.8	53.3	42.3	64.0	61.2	75.5	80.4
CV	1.81	1.33	1.46	1.30	1.32	1.57	1.33	1.55	1.85	1.85	1.54

CV: coefficient of variation; DDD: defined daily doses; Max.: maximum; Min.: minimum; N: number of participating partners providing data; Q: quarter; SD: standard deviation.

The codes after each antibiotic refer to the ATC code. The mandatory surveillance period spanned from the first quarter of 2018 to the fourth quarter of 2019.

### Resistance incidence in the hospital sector

Descriptive statistics of the quarterly incidence densities of resistant bacteria under study in HS are reported in Table 2. Pooled data analysis showed that ESBL-*E. coli* presented the highest level of incidence in HS (0.568 ± 0.045 cases/1,000 OBD per quarter) during the overall 2018─19 surveillance period, followed by ESBL-*K. pneumoniae* (0.418 ± 0.049), MRSA (0.256 ± 0.029), CR-*P. aeruginosa* (0.214 ± 0.032), CPE (0.126 ± 0.020), VRE (0.123 ± 0.026) and CR-*A. baumannii* (0.075 ± 0.021).

**Table 2:** Incidence density of resistant bacteria in clinical isolates in the hospital sector per 1,000 occupied bed-days per quarter, seven European countries, September 2017–May 2020 (n = 11 institutions)

Resistant bacteria	Quarters										
	2017	2018				2019				2020	
	Q4	Q1	Q2	Q3	Q4	Q1	Q2	Q3	Q4	Q1	Q2
Carbapenemase-producing Enterobacterales											
N	4	10	10	10	10	10	10	8	7	3	2
Mean	0.454	0.243	0.213	0.249	0.229	0.235	0.239	0.269	0.146	0.101	0.142
SD	0.830	0.383	0.306	0.319	0.339	0.329	0.324	0.375	0.219	0.121	0.091
Min.	0.000	0.000	0.000	0.000	0.009	0.000	0.000	0.000	0.000	0.000	0.077
Max.	1.698	1.226	0.958	0.953	1.038	0.995	0.892	1.034	0.615	0.235	0.206
CV	1.83	1.58	1.44	1.28	1.48	1.40	1.36	1.39	1.50	1.20	0.64
Extended-spectrum beta-lactamase-producing *Escherichia coli*											
N	3	9	9	9	9	9	9	7	6	3	2
Mean	0.541	0.666	0.700	0.738	0.686	0.775	0.818	0.860	0.953	0.874	0.914
SD	0.270	0.247	0.420	0.478	0.441	0.433	0.510	0.536	0.647	0.581	0.887
Min.	0.308	0.346	0.209	0.129	0.146	0.332	0.375	0.415	0.351	0.286	0.287
Max.	0.837	0.997	1.530	1.597	1.573	1.529	1.832	1.761	1.805	1.447	1.541
CV	0.50	0.37	0.60	0.65	0.64	0.56	0.62	0.62	0.68	0.66	0.97
Extended-spectrum beta-lactamase-producing *Klebsiella pneumoniae*											
N	3	9	9	9	9	9	9	7	6	3	2
Mean	0.636	0.511	0.477	0.549	0.508	0.545	0.613	0.409	0.353	0.283	0.358
SD	0.318	0.366	0.352	0.351	0.257	0.385	0.494	0.239	0.196	0.260	0.272
Min.	0.274	0.093	0.121	0.171	0.215	0.157	0.147	0.131	0.096	0.093	0.165
Max.	0.868	1.123	1.126	1.154	0.922	1.277	1.511	0.754	0.656	0.579	0.550
CV	0.50	0.72	0.74	0.64	0.51	0.71	0.81	0.58	0.55	0.92	0.76
Carbapenem-resistant *Acinetobacter baumannii*											
N	4	10	10	10	10	10	10	8	7	3	2
Mean	0.322	0.174	0.197	0.179	0.149	0.202	0.208	0.212	0.049	0.015	0.021
SD	0.318	0.275	0.361	0.271	0.230	0.369	0.352	0.415	0.099	0.019	0.030
Min.	0.006	0.000	0.000	0.000	0.000	0.000	0.000	0.000	0.000	0.000	0.000
Max.	0.698	0.868	1.136	0.810	0.662	1.168	1.110	1.202	0.269	0.036	0.042
CV	0.99	1.58	1.83	1.51	1.54	1.83	1.70	1.96	2.03	1.29	1.41
Carbapenem-resistant *Pseudomonas aeruginosa*											
N	4	10	10	10	10	10	10	8	7	3	2
Mean	0.110	0.353	0.329	0.475	0.360	0.429	0.353	0.380	0.325	0.626	0.663
SD	0.080	0.308	0.250	0.498	0.310	0.396	0.269	0.328	0.357	0.494	0.798
Min.	0.023	0.067	0.042	0.043	0.000	0.014	0.042	0.068	0.043	0.057	0.098
Max.	0.202	0.967	0.819	1.650	0.901	1.359	0.890	0.825	1.018	0.946	1.227
CV	0.73	0.87	0.76	1.05	0.86	0.92	0.76	0.86	1.10	0.79	1.21
Meticillin-resistant *Staphylococcus aureus*											
N	4	10	10	10	10	11	11	9	8	4	2
Mean	0.211	0.386	0.366	0.433	0.364	0.413	0.424	0.450	0.370	0.409	0.402
SD	0.149	0.229	0.264	0.301	0.270	0.268	0.328	0.402	0.366	0.382	0.320
Min.	0.079	0.115	0.051	0.068	0.098	0.045	0.026	0.031	0.061	0.024	0.175
Max.	0.419	0.734	0.842	0.967	0.817	0.844	0.927	1.106	0.919	0.782	0.628
CV	0.71	0.59	0.72	0.70	0.74	0.65	0.77	0.89	0.99	0.93	0.80
Vancomycin-resistant Enterococci (*E. faecalis and E. faecium*)											
N	4	10	10	10	10	10	11	8	7	3	2
Mean	0.152	0.144	0.137	0.178	0.157	0.302	0.221	0.165	0.114	0.004	0.007
SD	0.123	0.193	0.182	0.205	0.151	0.395	0.264	0.189	0.142	0.004	0.009
Min.	0.004	0.000	0.000	0.000	0.000	0.000	0.000	0.000	0.000	0.000	0.000
Max.	0.256	0.541	0.587	0.560	0.375	1.261	0.706	0.481	0.303	0.008	0.013
CV	0.81	1.34	1.33	1.15	0.96	1.31	1.19	1.15	1.25	1.00	1.41

CV: coefficient of variation; *E: Enterococcus*; Max.: maximum; Min.: minimum; N: number of participating partners providing data; SD: standard deviation.

The mandatory surveillance period spanned from the first quarter of 2018 to the fourth quarter of 2019.

A high level of variability was found between partners for the incidence densities of the resistant bacteria under surveillance in HS, with CVs for the 2018–19 surveillance period ranging from 0.58 for ESBL-*E. coli*, 0.67 for ESBL-*K. pneumoniae*, 0.73 for MRSA, 0.89 for CR-*P. aeruginosa*, with VRE (1.27); CPE (1.37); and CR-*A. baumannii* (1.73) showing the highest rates of variability (p < 0.0001).

### Antibiotic use in primary care

Table 3 depicts the descriptive statistics of the quarterly use of antibiotics in the community. During the 2018–19 surveillance period, overall antibiotic use in PC showed lower rates of variability compared with HS (CV: 0.26 in PC vs 0.35 in HS; p = 0.009). The three groups of antibiotics under study in PC showed similar CVs between participating institutions: 0.45 for amoxicillin/clavulanic acid, 0.45 for fluoroquinolones and 0.42 for macrolides (p = 0.81).

**Table 3:** Antibiotic use in the community in defined daily doses per 1,000 inhabitants per quarter, five European countries, September 2017 to May 2020 (n = 10 institutions)

Antibiotic	Quarters											
	2017	2018					2019				2020	
	Q4	Q1		Q2	Q3	Q4	Q1	Q2	Q3	Q4	Q1	Q2
Total antibiotics for systemic use (J01)												
N	5	10		10	10	10	10	10	10	10	5	3
Mean	19.3	21.6		17.3	14.3	16.2	16.6	13.3	12.0	14.4	15.8	9.0
SD	3.7	4.2		2.6	3.0	3.6	3.6	2.3	1.9	2.9	3.1	2.3
Min.	14.7	15.9		14.0	9.8	8.5	10.2	9.5	9.0	8.9	12.1	7.4
Max.	25.0	29.4		22.6	18.7	20.9	21.4	17.5	14.7	18.4	20.1	11.6
CV	0.19	0.19		0.15	0.21	0.22	0.22	0.18	0.16	0.20	0.20	0.25
Amoxicillin/clavulanic acid (J01CR02)												
N	5		10	10	10	10	10	10	10	10	5	3
Mean	6.2		7.8	6.5	5.5	5.7	5.0	4.1	4.0	4.3	3.8	3.2
SD	3.4		3.0	2.5	2.4	2.3	2.0	1.5	1.6	1.7	2.0	0.8
Min.	1.1		1.3	0.9	0.8	0.9	0.7	0.6	0.5	0.6	0.7	2.6
Max.	9.8		12.1	9.1	8.2	9.4	7.8	5.8	5.7	6.9	6.2	4.1
CV	0.55		0.39	0.38	0.43	0.40	0.40	0.37	0.40	0.41	0.52	0.27
Fluoroquinolones (J01MA)												
N	5		10	10	10	10	10	10	10	10	5	3
Mean	1.5		2.0	1.5	1.3	1.4	1.5	1.2	1.1	1.3	1.5	1.0
SD	1.0		0.9	0.6	0.5	0.6	0.7	0.5	0.4	0.5	0.7	0.3
Min.	0.4		0.5	0.4	0.4	0.4	0.4	0.3	0.4	0.4	0.4	0.8
Max.	2.7		3.5	2.3	2.0	2.3	2.7	2.0	1.6	2.2	2.4	1.3
CV	0.66		0.47	0.41	0.37	0.40	0.44	0.43	0.35	0.40	0.50	0.26
Macrolides (J01FA)												
N	5		10	10	10	10	10	10	10	10	5	3
Mean	1.9		2.5	1.8	1.4	2.0	2.4	1.7	1.4	1.9	2.0	0.8
SD	0.7		1.1	0.6	0.5	0.6	0.9	0.6	0.6	0.7	0.7	0.2
Min.	1.0		1.1	0.9	0.8	0.9	1.0	0.9	0.7	1.0	1.1	0.7
Max.	2.9		4.5	2.9	2.3	3.0	4.3	2.8	2.6	3.2	3.0	1.0
CV	0.35		0.42	0.35	0.35	0.31	0.39	0.33	0.43	0.35	0.35	0.18

CV: coefficient of variation; Max.: maximum; Min.: minimum; N: number of participating partners providing data; Q: quarter; SD: standard deviation.

The codes after each antibiotic refer to the ATC code. The mandatory surveillance period spanned from the first quarter of 2018 to the fourth quarter of 2019.

### Resistance incidence in primary care

Descriptive statistics of the quarterly incidence densities of resistant bacteria under study in PC are reported in Table 4. Pooled analysis indicated that ciprofloxacin-resistant *E. coli* showed the highest level of incidence in PC (0.448 ± 0.027 cases/1,000 inhabitants per quarter) during the surveillance period, followed by ESBL-*E. coli* (0.153 ± 0.008), ESBL-*K. pneumoniae* (0.054 ± 0.010), MRSA (0.049 ± 0.002) and CPE (0.003 ± 0.001).

**Table 4:** Incidence density of resistant bacteria in clinical isolates in the community per 1,000 inhabitants per quarter, four European countries, September 2017 to May 2020 (n = 7 institutions)

Resistant bacteria	Quarters										
	2017	2018				2019				2020	
	Q4	Q1	Q2	Q3	Q4	Q1	Q2	Q3	Q4	Q1	Q2
Carbapenemase-producing Enterobacterales											
N	3	6	5	6	6	6	6	6	6	3	1
Mean	0.015	0.014	0.017	0.003	0.003	0.003	0.004	0.004	0.004	0.000	0.001
SD	0.016	0.027	0.032	0.005	0.004	0.005	0.006	0.005	0.007	0.001	NA
Min.	0.000	0.000	0.000	0.000	0.000	0.000	0.000	0.000	0.000	0.000	0.001
Max.	0.032	0.067	0.074	0.013	0.011	0.012	0.015	0.012	0.017	0.001	0.001
CV	1.06	1.95	1.88	1.81	1.61	1.53	1.57	1.24	1.50	1.73	NA
Ciprofloxacin-resistant *Escherichia coli*											
N	3	7	7	7	7	7	7	7	7	4	2
Mean	0.417	0.790	0.863	0.661	0.460	0.429	0.540	0.480	0.437	0.631	0.391
SD	0.182	0.662	0.853	0.537	0.250	0.347	0.298	0.270	0.189	0.374	0.083
Min.	0.208	0.215	0.194	0.071	0.120	0.000	0.074	0.060	0.145	0.234	0.332
Max.	0.541	2.138	2.694	1.684	0.861	0.948	0.941	0.859	0.611	1.117	0.450
CV	0.44	0.84	0.99	0.81	0.54	0.81	0.55	0.56	0.43	0.59	0.21
Extended-spectrum beta-lactamase-producing *Escherichia coli*											
N	3	6	6	6	6	6	6	6	6	3	2
Mean	0.106	0.512	0.932	0.528	0.366	0.750	0.595	1.198	0.806	0.187	0.100
SD	0.061	0.803	1.854	0.900	0.485	1.448	1.031	2.548	1.585	0.071	0.018
Min.	0.039	0.078	0.078	0.046	0.088	0.067	0.085	0.060	0.095	0.144	0.087
Max.	0.159	2.138	4.714	2.357	1.347	3.704	2.694	6.397	4.040	0.269	0.112
CV	0.58	1.57	1.99	1.70	1.32	1.93	1.73	2.13	1.97	0.38	0.18
Extended-spectrum beta-lactamase-producing *Klebsiella pneumoniae*											
N	3	6	6	6	6	6	6	6	6	3	2
Mean	0.042	0.269	0.087	0.090	0.150	0.088	0.036	0.480	0.154	0.037	0.032
SD	0.025	0.567	0.123	0.122	0.257	0.123	0.023	1.085	0.254	0.012	0.018
Min.	0.027	0.019	0.027	0.021	0.021	0.014	0.000	0.021	0.032	0.028	0.019
Max.	0.070	1.426	0.337	0.337	0.673	0.337	0.071	2.694	0.673	0.051	0.044
CV	0.59	2.11	1.42	1.35	1.71	1.39	0.64	2.26	1.65	0.32	0.56
Meticillin-resistant *Staphylococcus aureus*											
N	4	7	7	7	7	7	7	7	7	4	2
Mean	0.040	0.039	0.034	0.036	0.035	0.042	0.039	0.045	0.043	0.055	0.027
SD	0.021	0.034	0.023	0.025	0.025	0.036	0.030	0.032	0.030	0.029	0.009
Min.	0.011	0.000	0.000	0.000	0.000	0.000	0.000	0.000	0.000	0.023	0.020
Max.	0.056	0.099	0.055	0.066	0.064	0.109	0.086	0.103	0.092	0.090	0.033
CV	0.51	0.87	0.68	0.70	0.72	0.87	0.77	0.71	0.69	0.53	0.35

CV: coefficient of variation; Max.: maximum; Min.: minimum; N: number of participating partners providing data; SD: standard deviation.

The mandatory surveillance period spanned from the first quarter of 2018 to the fourth quarter of 2019.

High levels of variability between partners were found, with CVs for the 2018–19 period ranging from 0.72 for MRSA; 0.81 for ciprofloxacin-resistant E. coli; 1.94 for ESBL-E. coli; 2.35 for CPE; and 2.62 for ESBL-K. pneumoniae (p < 0.0001).

### Barriers and needs

Once the pilot study came to an end, over half (9/17) of the partners expressed interest in continuing this quarterly surveillance in their hospitals/PC centres. Several barriers to achieving this periodicity in surveillance over time were reported by the participating institutions: (i) lack of IT infrastructure to collect data, (ii) lack of human resources dedicated to this task, (iii) insufficient institutional support to engage all stakeholders, (iv) high variability among the different health systems of the participating countries, (v) differences in indicators among countries that hamper the application and representativeness of surveillance data at a EU level.

## Discussion

In this work, we have reported the piloting and outcomes of a new AMR and AMU surveillance approach, a proof of concept for developing a quarterly surveillance system in the EU/EEA under the umbrella of the EU-JAMRAI joint action. Current data surveillance periodicity in the EU for both AMR and AMU is annual [14,15]. 

An innovative feature of the present approach is the quarterly frequency of data collection. AMR is a continuously evolving phenomenon that is affected by the use of antimicrobials over time [27]. In 2015, WHO highlighted the need of information on AMR incidence – among other outcomes – in a timely manner in order to guide antibiotic treatment, to inform local, regional and national actions and to assess the effectiveness of health interventions [9]. Quarterly surveillance is a feasible goal for delivering information promptly and in a manageable fashion [17-20]. Moreover, this type of system is especially useful if the data are publicly available, not anonymised and provide regular feedback to stimulate benchmarking and allow deviations to be corrected by the antimicrobial stewardship teams in every healthcare centre or healthcare system [20]. Our system would be complementary to EARS-Net and ESAC-Net and used to strengthen the knowledge on the evolution of AMR and AMU from the local to the regional and national level. This would provide data to inform interventions in a timelier manner.

This pilot system introduced new indicators to increase the knowledge of AMR and AMU status, allowing each healthcare centre to monitor their own data over time. The first is an AMU indicator for hospitals to assess the use in DDD per 1,000 bed-days, complementing the current EU/EEA AMU surveillance data for HS that are expressed as DDD per 1,000 inhabitants. Although census population as a denominator is appropriate to assess antibiotic use at the level of a country, province or large region, the estimation of AMU expressed as a rate with bed-days as the denominator provides a more accurate assessment of the use of antimicrobials in every healthcare centre, allowing benchmarking between similar services or centres in different institutions that may yield useful information [28]. By using this indicator, we found in our study that colistin was the antibiotic that showed the highest rate of variation between hospitals, proving to be an outcome which could help decision-makers prioritise interventions.

The second is an AMR indicator to assess the incidence density of resistant isolates from all clinical samples per 1,000 bed-days in HS, and per 1,000 inhabitants in PC. This measurement intended to complement the current EU/EEA AMR surveillance data that are expressed as resistance percentage from invasive (i.e. blood and cerebrospinal fluid) isolates [14]. The clinical relevance of indicator bacteria isolated from these samples is undisputable and prevents some of the inconsistencies that arise from differences in clinical case definitions, different sampling frames or heterogeneous healthcare utilisation. However, it should be noted that invasive isolates might not be representative of isolates of the same bacterial species from other type of common infections like urinary tract infections, respiratory tract infections and skin infections. Although resistance percentage is a very useful indicator for the clinician to choose the most adequate antibiotic for the individual patient, our proposed systematic surveillance of the incidence density indicator would be highly valuable to evaluate the ecological impact of control measures including those aimed to reduce the antibiotic pressure, as well as timely detection of outbreaks [29].

We piloted our study in selected countries with different healthcare systems and resources aiming to assess the feasibility of establishing a quarterly surveillance system for AMU and AMR. This feature imposed several limitations on our study that do not allow us to make any inference of the outcomes at the European level but rather consider this surveillance tool as a proof of concept. Firstly, the population coverage varied among partners, with some countries reporting data from large national surveillance systems, whereas other countries reported data from a smaller subset of local laboratories and hospitals or community healthcare areas. However, the indicators provided to the surveillance system were intended to be easily produced by merging all the outcomes provided in a bottom-up approach by the participating HS/PC centres in a region or country, allowing both the benefit of monitoring their own levels of AMU and AMR at each centre and the gradual engagement of participants into the system with the resulting aggregated data. Secondly, differences in the organisation of healthcare systems and the specificity of groups of patients in different care sectors between countries should be taken into consideration, as well as the fact that not all the participating institutions considered the same hospital departments for AMU reporting. This would underline the strength of the surveillance system to first promote self-assessment, while benchmarking of AMU should be taken with caution when comparing different healthcare systems. Thirdly, AMU data were reported following the ATC-WHO-2018 DDD calculations for the quarters of 2017 and 2018 and data for the quarters of 2019 and 2020 were reported following the new ATC-WHO-2019 calculations. Therefore, because of this change of measurement units during the piloting surveillance period, trends could not be used to compare the evolution of antibiotic use between 2018 and 2019. In spite of this issue, the quarterly frequency of the surveillance proved to be a valuable tool for the early detection of increased use of antibiotics in hospitals and a decreased use of antibiotics in the community. This was illustrated by those institutions reporting data for the second quarter of 2020 concurrently with the coronavirus disease (COVID-19) pandemic, compared with the same quarter of 2019, an observation that further studies have recently confirmed [30-33]. Because of the observed variability among the different participating institutions, descriptive analyses shown must be taken with caution when making any inference and cannot be taken as representative of AMU or AMR in the EU/EEA or when comparing outcomes with the 2018–19 EARS-Net data [34]. Fourthly, because of the observed variability among the different participating institutions, no trend analysis of aggregated AMR data could be conducted. However, the advantage of collecting data on a quarterly basis for the participating institutions would allow them to analyse their own time series in short periods of time like that presented in our study; this could be done in every healthcare centre as well as in merged data to help identify their own slopes and possible changes in trends sooner. Finally, the quarterly concept required that participant institutions would provide quarterly data by the end of the following quarter (data from Q1 submitted by end of Q2, i.e. within 3 months). Our findings show that only ca one third of the partners were able to do so. Even considering that all participants expected to meet the data submission timeline, several partners found barriers during the development of the pilot study that hindered collecting and submitting their quarterly data, a fact that occurred both in partners participating with one single centre and in partners reporting data at a regional/national level.

Although half of the partners expressed interest in continuing the quarterly monitoring once the pilot study was completed, the barriers and needs identified by the participants should be first addressed before implementation to improve the feasibility of this surveillance approach: modern and integrated IT systems, to surpass the reported lack of IT infrastructure to collect data; sufficient manpower to collect, merge and analyse AMU and AMR data, to overcome the low amount of human resources dedicated to this task; committed institutional support to engage all stakeholders, with better institutional coordination between microbiological and AMU data providers; capacity to adapt the system to the high variability among the different health systems of the participating countries; and harmonisation of indicators to overcome the differences among countries that hamper the application and representativeness of surveillance data at a EU level.

## Conclusion

The results of our pilot study indicate that this could be a first step towards the development of a quarterly surveillance system for AMU and AMR in both HS and PC in the EU/EEA. However, there are some caveats that should be addressed before implementation of a system to collect data and provide outcomes within shorter time periods than annually at the European level. These include committed institutional support, dedicated human resources and coordination of microbiological and AMU data sources, which requires more homogeneous indicators and modern integrated IT systems.

## EU-JAMRAI WP7.4.1 group members

Austria: Vera Buhmann, Anton Hlava, (Austrian Public Health Institute, Vienna, Austria); Croatia: Blaženka Hunjak, Pero Ivanko, Anamarija Škoda, (Croatian Institute of Public Health, Zagreb, Croatia); Czech Republic: Helena Žemličková, Barbora Mackova, (The National Institute of Public Health, Prague, Czech Republic); Denmark: Sissel Skovgaard, Ute Wolff Sönksen, (Statens Serum Institut, Copenhagen, Denmark); Germany: Arina Zanuzdana, Tim Eckmanns, (Robert Koch Institute, Berlin, Germany); Greece: Alkiviadis Vatopoulos, Michalis Polemis, (Ethniki Scholi Dimosias Ygeias - National School of Public Health, Athens, Greece); Italy: Domenico Martinelli, Francesca Fortunato, (University of Foggia, Foggia, Italy); Lithuania: Asta Dambrauskiene, (The Hospital of Lithuanian University of Health Sciences Kauno Klinikos, Kaunas, Lithuania); Lithuania: Rolanda Valinteliene, (Institute of Hygiene, Vilnius, Lithuania); Poland: Anna Olczak-Pieńkowska, Dorota Żabicka, (The National Medicines Institute, Warsaw, Poland); Portugal: Maria Rodrigues, Nuno Pereira, Isabel Neves, (Directorate-General of Health, Lisbon, Portugal); Spain – Andalusia: José Antonio Lepe, Raquel Valencia, María Victoria Gil-Navarro, (Institute of Biomedicine of Seville, Andalusian Health Service, Seville, Spain); Spain - Balearic Islands: Antonio Oliver-Palomo, Leonor del Mar Periañez-Parraga, Estrella Rojo-Molinero, (Institut D’investigació Sanitària Illes Balears (Idisba), Conselleria de Salud del Govern de les Illes Balears, Palma De Mallorca, Spain); Spain – Catalonia: Glòria Oliva, Marta Massanes, (Dirección General de Ordenación y Regulación Sanitaria, Departamento de Salud de la Generalitat de Cataluña, Barcelona, Spain); Spain – Madrid: José Manuel Izquierdo-Palomares, Ainhoa Aranguren, (Dirección General de Coordinación de La Asistencia Sanitaria, Servicio Madrileño de Salud, Madrid, Spain); Spain – Murcia: Víctor José Rausell-Rausell, María del Pilar López-Acuña, (Servicio Murciano de Salud (SMS) - Fundación para la Formación e Investigaciones Sanitarias de la Región de Murcia (FFIS), Murcia, Spain); Spain – Navarre: Alberto Gil-Setas, María Eugenia Portillo, (Fundación Miguel Servet – Navarrabiomed, Departamento de Salud del Gobierno de Navarra, Pamplona, Spain).

## Supplementary Data

6,778,000 Supplement
