# Supplementary material for: A step forward in antibiotic use and resistance monitoring: a quarterly surveillance system pilot in 11 European Union/European Economic Area countries, September 2017 to May 2020
Source: Euro Surveill. 2022 Nov 17;27(46):2200082. doi: 10.2807/1560-7917.ES.2022.27.46.2200082 (PMC9673239; doi:10.2807/1560-7917.ES.2022.27.46.2200082)
Supplement: Supplement [file 22-00082_CISNEROS_SUPPLEMENT.pdf]

SUPPLEMENTARY MATERIAL

This supplementary material is hosted by Eurosurveillance as supporting information alongside the article ‘A step forward in monitoring antibiotic use and antimicrobial resistance: a quarterly surveillance pilot approach in Europe, 11 European countries, September 2017 to May 2020’, on behalf of the authors, who remain responsible for the accuracy and appropriateness of the content. The same standards for ethics, copyright, attributions and permissions as for the article apply. Supplements are not edited by Eurosurveillance and the journal is not responsible for the maintenance of any links or email addresses provided therein.

TABLE S1. Participating institutions in Task 7.4.1

Code	Country/Autonomous Community	Institution	Acronym
AT-3	Austria	Austrian Public Health Institute	GOG
HR-6	Croatia	Croatian Institute of Public Health	CIPH-HZJZ
CZ-7	Czech Republic	The National Institute of Public Health	NIPH
DK-8	Denmark	Statens Serum Institut	SSI
DE-10	Germany	Robert Koch-Institute	RKI
GR-12	Greece	Ethniki Scholi Dimosias Ygeias - National School of Public Health	ESDY NSPH
IT-14	Italy	University of Foggia	UNIFG
LT-17	Lithuania	The Hospital of Lithuanian University of Health Sciences Kauno Klinikos	LSMULKK
LT-19	Lithuania	Institute of Hygiene	HI
PL-25	Poland	The National Medicines Institute	NMI
PT-26	Portugal	Directorate-General of Health	DGS
SP-30	Spain - Catalonia	Departamento de Salud de la Generalitat de Cataluña	GENCAT
SP-31	Spain - Balearic Islands	Conselleria de Salud del Govern de les Illes Balears	IdISBa
SP-32	Spain - Murcia	Servicio Murciano de Salud - Fundación para la Formación e Investigaciones Sanitarias de la Región de Murcia	SMS FFIS
SP-33	Spain - Navarre	Fundación Miguel Servet - Navarrabiomed	FMS
SP-34	Spain - Andalusia	Servicio Andaluz de Salud - FISEVI	SAS
SP-36	Spain - Madrid	Servicio Madrileño de Salud	SERMAS

FIGURE S1. Data collection form for the hospital sector

EU-JAMRAI. Near real time surveillance of antimicrobials and multidrug resistant bacteria in human medicine

Data collection spreadsheet

	Data from
	HOSPITALS

Country:
Region: <- Fill in case of regional aggregated data
Hospital(s): <- Fill in case of data from one o more selected hospitals

Year:	2020	
Trimester:	4th QUARTER	(OCT-NOV-DEC / 2020)

Type	Indicator	Definition	Numerator	Denominator	Outcome
Hospitals Antimicrobial Consumption	Overall DDD/1000 OBD of antibiotics	Total DDD of antibiotics (J01) a trimester x 1000/No. of total stays during the trimester			#DIV/0!
Hospitals Antimicrobial Consumption	Overall DDD/1000 OBD of antifungals (optional)	Total DDD of antifungals (J02) a trimester x 1000/No. of total stays during the trimester			#DIV/0!
Hospitals Antimicrobial Consumption	Overall DDD/1000 OBD of antimicrobials (optional)	Total DDD of antibiotic (J01) and antifungals (J02) a trimester x 1000/No. of total stays during the trimester			#DIV/0!
Hospitals Antimicrobial Consumption	DDD/1000 OBD of piperacillin-tazobactam	Consumption (grams) of piperacillin-tazobactam a trimester x 1000/DDD of this antimicrobial drug x No. of total stays during the trimester			#DIV/0!
Hospitals Antimicrobial Consumption	DDD/1000 OBD of amoxicillin-clavulanate	Consumption (grams) of amoxicillin-clavulanate a trimester x 1000/DDD of this antimicrobial drug x No. of total stays during the trimester			#DIV/0!
Hospitals Antimicrobial Consumption	DDD/1000 OBD of ertapenem	Consumption (grams) of ertapenem a trimester x 1000/DDD of this antimicrobial drug x No. of total stays during the trimester			#DIV/0!
Hospitals Antimicrobial Consumption	DDD/1000 OBD of imipenem	Consumption (grams) of imipenem a trimester x 1000/DDD of this antimicrobial drug x No. of total stays during the trimester			#DIV/0!
Hospitals Antimicrobial Consumption	DDD/1000 OBD of meropenem	Consumption (grams) of meropenem a trimester x 1000/DDD of this antimicrobial drug x No. of total stays during the trimester			#DIV/0!
Hospitals Antimicrobial Consumption	DDD/1000 OBD of doripenem	Consumption (grams) of doripenem a trimester x 1000/DDD of this antimicrobial drug x No. of total stays during the trimester			#DIV/0!
Hospitals Antimicrobial Consumption	DDD/1000 OBD of carbapenems		0.00	0	#DIV/0!
Hospitals Antimicrobial Consumption	DDD/1000 OBD of ceftriaxone	Consumption (grams) of ceftriaxone a trimester x 1000/DDD of this antimicrobial drug x No. of total stays during the trimester			#DIV/0!
Hospitals Antimicrobial Consumption	DDD/1000 OBD of cefotaxime	Consumption (grams) of cefotaxime a trimester x 1000/DDD of this antimicrobial drug x No. of total stays during the trimester			#DIV/0!
Hospitals Antimicrobial Consumption	DDD/1000 OBD of ceftazidime	Consumption (grams) of ceftazidime a trimester x 1000/DDD of this antimicrobial drug x No. of total stays during the trimester			#DIV/0!
Hospitals Antimicrobial Consumption	DDD/1000 OBD of cefepime	Consumption (grams) of cefepime a trimester x 1000/DDD of this antimicrobial drug x No. of total stays during the trimester			#DIV/0!
Hospitals Antimicrobial Consumption	DDD/1000 OBD of 3rd and 4th cephalosporins		0.00	0	#DIV/0!
Hospitals Antimicrobial Consumption	DDD/1000 OBD of ciprofloxacin	Consumption (grams) of ciprofloxacin a trimester x 1000/DDD of this antimicrobial drug x No. of total stays during the trimester			#DIV/0!
Hospitals Antimicrobial Consumption	DDD/1000 OBD of levofloxacin	Consumption (grams) of levofloxacin a trimester x 1000/DDD of this antimicrobial drug x No. of total stays during the trimester			#DIV/0!
Hospitals Antimicrobial Consumption	DDD/1000 OBD of moxifloxacin	Consumption (grams) of moxifloxacin a trimester x 1000/DDD of this antimicrobial drug x No. of total stays during the trimester			#DIV/0!
Hospitals Antimicrobial Consumption	DDD/1000 OBD of quinolones		0.00	0	#DIV/0!
Hospitals Antimicrobial Consumption	DDD/1000 OBD of vancomycin	Consumption (grams) of vancomycin a trimester x 1000/DDD of this antimicrobial drug x No. of total stays during the trimester			#DIV/0!
Hospitals Antimicrobial Consumption	DDD/1000 OBD of colistin	Consumption (grams) of colistin a trimester x 1000/DDD of this antimicrobial drug x No. of total stays during the trimester			#DIV/0!
Hospitals AntiMicrobial Resistance	Incidence density of inpatients with carbapenemase-producing Enterobacteriaceae (CPE)	No. of inpatients with CPE infection/colonisation x 1000/No. of total stays during the trimester			#DIV/0!
Hospitals AntiMicrobial Resistance	Incidence density of inpatients with ESBL *Escherichia coli*	No. of inpatients with ESBL *E. coli* infection/colonisation x 1000/No. of total stays during the trimester			#DIV/0!
Hospitals AntiMicrobial Resistance	Incidence density of inpatients with ESBL *Klebsiella pneumoniae*	No. of inpatients with ESBL *K. pneumoniae* infection/colonisation x 1000/No. of total stays during the trimester			#DIV/0!
Hospitals AntiMicrobial Resistance	Incidence density of inpatients with carbapenem-resistant *Acinetobacter baumannii*	No. of inpatients with multidrug-resistant *A. baumannii* infection/colonisation x 1000/No. of total stays during the trimester			#DIV/0!
Hospitals AntiMicrobial Resistance	Incidence density of inpatients with carbapenem-resistant *Pseudomonas aeruginosa*	No. of inpatients with multidrug-resistant *P. aeruginosa* infection/colonisation x 1000/No. of total stays during the trimester			#DIV/0!
Hospitals AntiMicrobial Resistance	Incidence density of inpatients with meticillin-resistant *Staphylococcus aureus*	No. of inpatients with methicillin-resistant *S. aureus* infection/colonisation x 1000/No. of total stays during the trimester			#DIV/0!
Hospitals AntiMicrobial Resistance	Incidence density of inpatients with vancomycin resistant enterococci	No. of inpatients with vancomycin resistant *Enterococcus faecalis and E. faecium* infection/colonisation x 1000/No. of total stays during the trimester			#DIV/0!

FIGURE S2. Data collection form for primary care

EU-JAMRAI. Near real time surveillance of antimicrobials and multidrug resistant bacteria in human medicine

Data collection spreadsheet

**Country:**	
**Region:**	<- Fill in case of regional aggregated data

Data from **PRIMARY CARE**

**Year:**	**2020**
**Trimester:**	**4th quarter**

(OCT-NOV-DEC / 2020) Days = **92**

Type	Indicator	Definition	Numerator	Denominator	Outcome
Community Antimicrobial Consumption	DID of antibiotics in primary care	Total DDD of antibiotic (**J01**) x 1000/ No. of inhabitants during the trimester			#DIV/0!
Community Antimicrobial Consumption	DID of antifungals in primary care (optional)	Total DDD of antifungals (**J02**) x 1000/ No. of inhabitants during the trimester			#DIV/0!
Community Antimicrobial Consumption	DID of overall antimicrobials in primary care (optional)	Total DDD of antibiotic (**J01**) and antifungals (**J02**) x 1000/ No. of inhabitants during the trimester			#DIV/0!
Community Antimicrobial Consumption	DID of amoxycillin-clavulanate	No. of packaging units sold of amoxycillin-clavulanate x No. of pharmaceutical forms per package x grams of active ingredient in each pharmaceutical form x 1000/DDD of this antimicrobial drug x No. of inhabitants during the trimester			#DIV/0!
Community Antimicrobial Consumption	DID of ciprofloxacin	No. of packaging units sold of ciprofloxacin x No. of pharmaceutical forms per package x grams of active ingredient in each pharmaceutical form x 1000/DDD of this antimicrobial drug x No. of inhabitants during the trimester			#DIV/0!
Community Antimicrobial Consumption	DID of levofloxacin	No. of packaging units sold of levofloxacin x No. of pharmaceutical forms per package x grams of active ingredient in each pharmaceutical form x 1000/DDD of this antimicrobial drug x No. of inhabitants during the trimester			#DIV/0!
Community Antimicrobial Consumption	DID of moxifloxacin	No. of packaging units sold of moxifloxacin x No. of pharmaceutical forms per package x grams of active ingredient in each pharmaceutical form x 1000/DDD of this antimicrobial drug x No. of inhabitants during the trimester			#DIV/0!
Community Antimicrobial Consumption	DID of quinolones		0.00	0	#DIV/0!
Community Antimicrobial Consumption	DID of erythromycin	No. of packaging units sold of erythromycin x No. of pharmaceutical forms per package x grams of active ingredient in each pharmaceutical form x 1000/DDD of this antimicrobial drug x No. of inhabitants during the trimester			#DIV/0!
Community Antimicrobial Consumption	DID of clarithromycin	No. of packaging units sold of clarithromycin x No. of pharmaceutical forms per package x grams of active ingredient in each pharmaceutical form x 1000/DDD of this antimicrobial drug x No. of inhabitants during the trimester			#DIV/0!
Community Antimicrobial Consumption	DID of azithromycin	No. of packaging units sold of azithromycin x No. of pharmaceutical forms per package x grams of active ingredient in each pharmaceutical form x 1000/DDD of this antimicrobial drug x No. of inhabitants during the trimester			#DIV/0!
Community Antimicrobial Consumption	DID of macrolides		0.00	0	#DIV/0!
Community AntiMicrobial Resistance	Incidence density of outpatients with carbapenemase-producing Enterobacteriaceae (CPE)	No. of outpatients with CPE infection/colonisation x 1000/No. of inhabitants during the trimester			#DIV/0!
Community AntiMicrobial Resistance	Incidence density of outpatients with ciprofloxacin-resistant *Escherichia coli*	No. of outpatients with ciprofloxacin-resistant *E. coli* infection/colonisation x 1000/No. of inhabitants during the trimester			#DIV/0!
Community AntiMicrobial Resistance	Incidence density of outpatients with ESBL *Escherichia coli*	No. of outpatients with ESBL *E. coli* infection/colonisation x 1000/No. of inhabitants during the trimester			#DIV/0!
Community AntiMicrobial Resistance	Incidence density of outpatients with ESBL *Klebsiella pneumoniae*	No. of outpatients with ESBL *K. pneumoniae* infection/colonisation x 1000/No. of inhabitants during the trimester			#DIV/0!
Community AntiMicrobial Resistance	Incidence density of outpatients with meticillin-resistant *Staphylococcus aureus*	No. of outpatients with methicillin-resistant *S. aureus* infection/colonisation x 1000/No. of inhabitants during the trimester			#DIV/0!

TABLE S2. Healthcare scope, geographical scope and surveillance coverage

Institution	Healthcare level		Geographical level			Quarterly surveillance coverage average	
	Hospital sector	Primary care	Local	Regional	National	Hospital stays	Inhabitants
AT-3	Y	-	Y	-	-	71596	NA
HR-6	-	Y	-	-	Y	NA	4100000
CZ-7	Y	-	Y	-	-	111632	NA
DK-8	Y	Y	-	-	Y	915917	5800000
DE-10	Y	-	-	-	Y	4927038	NA
GR-12	Y	-	Y	-	-	44316	NA
IT-14	-	Y	Y	-	-	NA	3000
LT-17	Y	-	Y	-	-	160443	NA
LT-19	Y	-	Y	-	-	40153	NA
PL-25	Y	-	Y	-	-	105373	NA
PT-26	Y	Y	-	-	Y	*	9800000
SP-30	Y	Y	-	Y	-	185064	7500000
SP-31	Y	Y	-	Y	-	128684	1100000
SP-32	Y	Y	-	Y	-	195108	1400000
SP-33	Y	Y	-	Y	-	73715	470000
SP-34	Y	Y	-	Y	-	853801	8200000
SP-36	Y	Y	-	Y	-	162616	6700000
Total	15	10	7	6	4	7975456	45073000

Local: 1 to 3 hospitals or 1 healthcare area; Regional: Region or Autonomous Community; Hospital stays: average hospital occupied bed-days covered per quarter; Inhabitants: average population covered per quarter; NA: not applicable.

* Antimicrobial use denominators are referred to inhabitants, not to hospital stays.

TABLE S3. Quarterly surveillance data reporting in the hospital sector

Code	Acronym	Data Type	2018 Q1	2018 Q2	2018 Q3	2018 Q4	2019 Q1	2019 Q2	2019 Q3	2019 Q4	2020 Q1	2020 Q2
AT-3	GOG	AMC	Y	Y	Y	Y	Y	Y	Y	Y	-	-
		AMR	Y	Y	Y	Y	Y	Y	Y	Y	-	-
HR-6	CIPH HZJZ	AMC	-	-	-	-	-	-	-	-	-	-
		AMR	-	-	-	-	-	-	-	-	-	-
CZ-7	NIPH	AMC	Y	Y	Y	Y	Y	Y	-	-	-	-
		AMR	Y	Y	Y	Y	Y	Y	-	-	-	-
DK-8	SSI	AMC	Y	Y	Y	Y	Y	Y	Y	Y	-	-
		AMR	Y	Y	Y	Y	Y	Y	Y	Y	-	-
DE-10	RKI	AMC	Y	Y	Y	Y	-	-	-	-	-	-
		AMR	-	-	-	-	-	-	-	-	-	-
GR-12	ESDY NSPH	AMC	Y	Y	Y	Y	Y	Y	Y	-	-	-
		AMR	Y	Y	Y	Y	Y	Y	Y	-	-	-
IT-14	UNIFG	AMC	-	-	-	-	-	-	-	-	-	-
		AMR	-	-	-	-	-	-	-	-	-	-
LT-17	LSMULKK	AMC	Y	Y	Y	Y	Y	Y	Y	Y	Y	-
		AMR	Y	Y	Y	Y	Y	Y	Y	Y	Y	-
LT-19	HI	AMC	Y	Y	Y	Y	Y	Y	Y	Y	Y	-
		AMR	-	-	-	-	Y	Y	Y	Y	Y	-
PL-25	NMI	AMC	Y	Y	Y	Y	Y	Y	Y	-	-	-
		AMR	Y	Y	Y	Y	Y	Y	Y	-	-	-
PT-26	DGS	AMC	Y	Y	Y	Y	Y	Y	Y	Y	-	-
		AMR	-	-	-	-	-	-	-	-	-	-
SP-30	GENCAT	AMC	Y	Y	Y	Y	Y	Y	Y	Y	-	-
		AMR	-	-	-	-	-	-	-	-	-	-
SP-31	IdISBa	AMC	Y	Y	Y	Y	Y	Y	Y	Y	Y	Y
		AMR	Y	Y	Y	Y	Y	Y	Y	Y	Y	Y
SP-32	SMS FFIS	AMC	Y	Y	Y	Y	Y	Y	Y	Y	Y	Y
		AMR	-	-	-	-	-	-	-	-	-	-
SP-33	FMS	AMC	Y	Y	Y	Y	Y	Y	Y	Y	Y	-
		AMR	Y	Y	Y	Y	Y	Y	Y	Y	Y	-
SP-34	SAS	AMC	Y	Y	Y	Y	Y	Y	Y	Y	Y	Y
		AMR	Y	Y	Y	Y	Y	Y	Y	Y	Y	Y
SP-36	SERMAS	AMC	Y	Y	Y	Y	Y	Y	Y	Y	-	-
		AMR	Y	Y	Y	Y	Y	Y	Y	Y	-	-

AMC: antimicrobial consumption; AMR: antimicrobial resistance; Y: data informed; (-): no data informed

TABLE S4. Indicators for the hospital sector provided per partner

**HOSPITAL CARE**																	
**Antimicrobial use indicators**	**AT-3**	**HR-6**	**CZ-7**	**DK-8**	**DE-10**	**GR-12**	**IT-14**	**LT-17**	**LT-19**	**PL-25**	**PT-26***	**SP-30**	**SP-31**	**SP-32**	**SP-33**	**SP-34**	**SP-36**
Total antibiotics	Y	-	Y	Y	Y	Y	-	Y	Y	Y	Y	Y	Y	Y	Y	Y	Y
Total antifungals (optional)	Y	-	Y	Y	Y	-	-	-	-	Y	-	Y	Y	Y	Y	Y	Y
Overall antimicrobials (optional)	Y	-	Y	Y	Y	-	-	-	-	Y	-	Y	Y	Y	Y	Y	Y
Piperacillin-tazobactam	Y	-	Y	Y	Y	Y	-	Y	Y	Y	Y	Y	Y	Y	Y	Y	Y
Amoxicillin-clavulanate	Y	-	Y	Y	Y	Y	-	Y	Y	Y	Y	Y	Y	Y	Y	Y	Y
Ertapenem	Y	-	Y	Y	Y	Y	-	Y	-	Y	Y	Y	Y	Y	Y	Y	Y
Imipenem	Y	-	Y	Y	Y	Y	-	Y	Y	Y	Y	Y	Y	Y	-	Y	Y
Meropenem	Y	-	Y	Y	Y	Y	-	Y	Y	Y	Y	Y	Y	Y	Y	Y	Y
Doripenem	Y	-	Y	Y	Y	Y	-	Y	-	Y	Y	Y	Y	Y	-	Y	Y
Sum of carbapenems	Y	-	Y	Y	Y	Y	-	Y	Y	Y	Y	Y	Y	Y	Y	Y	Y
Ceftriaxone	Y	-	Y	Y	Y	Y	-	Y	Y	Y	Y	Y	Y	Y	Y	Y	Y
Cefotaxime	Y	-	Y	Y	Y	Y	-	Y	Y	Y	Y	Y	Y	Y	Y	Y	Y
Ceftazidime	Y	-	Y	Y	Y	Y	-	Y	Y	Y	Y	Y	Y	Y	Y	Y	Y
Cefepime	Y	-	Y	Y	Y	Y	-	Y	-	Y	Y	Y	Y	Y	Y	Y	Y
Sum of 3rd and 4th cephalosporins	Y	-	Y	Y	Y	Y	-	Y	Y	Y	Y	Y	Y	Y	Y	Y	Y
Ciprofloxacin	Y	-	Y	Y	Y	Y	-	Y	Y	Y	Y	Y	Y	Y	Y	Y	Y
Levofloxacin	Y	-	Y	Y	Y	Y	-	Y	-	Y	Y	Y	Y	Y	Y	Y	Y
Moxifloxacin	Y	-	Y	Y	Y	Y	-	Y	-	Y	Y	Y	Y	Y	-	Y	Y
Sum of quinolones	Y	-	Y	Y	Y	Y	-	Y	Y	Y	Y	Y	Y	Y	Y	Y	Y
Vancomycin	Y	-	Y	Y	Y	Y	-	Y	Y	Y	Y	Y	Y	Y	Y	Y	Y
Colistin	Y	-	Y	Y	Y	Y	-	Y	-	Y	Y	Y	Y	Y	Y	Y	Y
AMC indicators under surveillance	100%	0%	100%	100%	100%	100%	0%	100%	68%	100%	100%	100%	100%	100%	84%	100%	100%

**HOSPITAL CARE**																	
**Antimicrobial resistance indicators**	**AT-3**	**HR-6**	**CZ-7**	**DK-8**	**DE-10**	**GR-12**	**IT-14**	**LT-17**	**LT-19**	**PL-25**	**PT-26**	**SP-30**	**SP-31**	**SP-32**	**SP-33**	**SP-34**	**SP-36**
CPE	Y	-	Y	Y	-	Y	-	Y	-	Y	-	-	Y	-	Y	Y	Y
ESBL *Escherichia coli*	Y	-	Y	-	-	Y	-	Y	-	Y	-	-	Y	-	Y	Y	Y
ESBL *Klebsiella pneumoniae*	Y	-	Y	-	-	Y	-	Y	-	Y	-	-	Y	-	Y	Y	Y
CR *Acinetobacter baumannii*	Y	-	Y	Y	-	Y	-	Y	-	Y	-	-	Y	-	Y	Y	Y
CR *Pseudomonas aeruginosa*	Y	-	Y	Y	-	Y	-	Y	-	Y	-	-	Y	-	Y	Y	Y
MRSA	Y	-	Y	Y	-	Y	-	Y	Y	Y	-	-	Y	-	Y	Y	Y
VRE	Y	-	Y	Y	-	Y	-	Y	-	Y	-	-	Y	-	Y	Y	Y
AMR indicators under surveillance	100%	0%	100%	71%	0%	100%	0%	100%	14%	100%	0%	0%	100%	0%	100%	100%	100%

Antimicrobial use (AMC) was assessed in DDD (defined daily doses) per 1000 OBD (occupied bed-days); Antimicrobial resistance (AMR) incidence density was assessed as number of inpatients with positive clinical isolates per 1000 OBD; CPE: Carbapenemase-producing Enterobacteriaceae; ESBL: Extended-spectrum beta-lactamase; CR: Carbapenem-resistant; MRSA: meticillin-resistant *Staphylococcus aureus*; VRE: Vancomycin resistant enterococci. (*) PT-26 antimicrobial use denominators are referred to inhabitants, not to hospital stays; Y: data informed; (-): no data informed.

TABLE S5. Quarterly surveillance data reporting in primary care

Code	Acronym	Data Type	2018 Q1	2018 Q2	2018 Q3	2018 Q4	2019 Q1	2019 Q2	2019 Q3	2019 Q4	2020 Q1	2020 Q2
AT-3	GOG	AMC	-	-	-	-	-	-	-	-	-	-
		AMR	-	-	-	-	-	-	-	-	-	-
HR-6	CIPH HZJZ	AMC	Y	Y	Y	Y	Y	Y	Y	Y	-	-
		AMR	Y	Y	Y	Y	Y	Y	Y	Y	-	-
CZ-7	NIPH	AMC	-	-	-	-	-	-	-	-	-	-
		AMR	-	-	-	-	-	-	-	-	-	-
DK-8	SSI	AMC	Y	Y	Y	Y	Y	Y	Y	Y	Y	-
		AMR	Y	Y	Y	Y	Y	Y	Y	Y	Y	-
DE-10	RKI	AMC	-	-	-	-	-	-	-	-	-	-
		AMR	-	-	-	-	-	-	-	-	-	-
GR-12	ESDY NSPH	AMC	-	-	-	-	-	-	-	-	-	-
		AMR	-	-	-	-	-	-	-	-	-	-
IT-14	UNIFG	AMC	Y	Y	Y	Y	Y	Y	Y	Y	-	-
		AMR	Y	Y	Y	Y	Y	Y	Y	Y	-	-
LT-17	LSMULKK	AMC	-	-	-	-	-	-	-	-	-	-
		AMR	-	-	-	-	-	-	-	-	-	-
LT-19	HI	AMC	-	-	-	-	-	-	-	-	-	-
		AMR	-	-	-	-	-	-	-	-	-	-
PL-25	NMI	AMC	-	-	-	-	-	-	-	-	-	-
		AMR	-	-	-	-	-	-	-	-	-	-
PT-26	DGS	AMC	Y	Y	Y	Y	Y	Y	Y	Y	-	-
		AMR	-	-	-	-	-	-	-	-	-	-
SP-30	GENCAT	AMC	Y	Y	Y	Y	Y	Y	Y	Y	-	-
		AMR	-	-	-	-	-	-	-	-	-	-
SP-31	IdISBa	AMC	Y	Y	Y	Y	Y	Y	Y	Y	Y	Y
		AMR	Y	Y	Y	Y	Y	Y	Y	Y	Y	Y
SP-32	SMS FFIS	AMC	Y	Y	Y	Y	Y	Y	Y	Y	Y	Y
		AMR	-	-	-	-	-	-	-	-	-	-
SP-33	FMS	AMC	Y	Y	Y	Y	Y	Y	Y	Y	Y	-
		AMR	Y	Y	Y	Y	Y	Y	Y	Y	Y	-
SP-34	SAS	AMC	Y	Y	Y	Y	Y	Y	Y	Y	Y	Y
		AMR	Y	Y	Y	Y	Y	Y	Y	Y	Y	Y
SP-36	SERMAS	AMC	Y	Y	Y	Y	Y	Y	Y	Y	-	-
		AMR	Y	Y	Y	Y	Y	Y	Y	Y	-	-

AMC: antimicrobial consumption; AMR: antimicrobial resistance; Y: data informed; (-): no data informed

TABLE S6. Indicators for primary care provided per partner

PRIMARY CARE																	
**Antimicrobial use indicators**	**AT-3**	**HR-6**	**CZ-7**	**DK-8**	**DE-10**	**GR-12**	**IT-14**	**LT-17**	**LT-19**	**PL-25**	**PT-26**	**SP-30**	**SP-31**	**SP-32**	**SP-33**	**SP-34**	**SP-36**
Total antibiotics	-	Y	-	Y	-	-	Y	-	-	-	Y	Y	Y	Y	Y	Y	Y
Total antifungals (optional)	-	-	-	Y	-	-	Y	-	-	-	-	Y	Y	Y	Y	Y	Y
Overall antimicrobials (optional)	-	-	-	Y	-	-	Y	-	-	-	-	Y	Y	Y	Y	Y	Y
Amoxicillin-clavulanate	-	Y	-	Y	-	-	Y	-	-	-	Y	Y	Y	Y	Y	Y	Y
Ciprofloxacin	-	Y	-	Y	-	-	Y	-	-	-	Y	Y	Y	Y	Y	Y	Y
Levofloxacin	-	Y	-	Y	-	-	Y	-	-	-	Y	Y	Y	Y	Y	Y	Y
Moxifloxacin	-	Y	-	Y	-	-	Y	-	-	-	Y	Y	Y	Y	Y	Y	Y
Sum of quinolones	-	Y	-	Y	-	-	Y	-	-	-	Y	Y	Y	Y	Y	Y	Y
Erythromycin	-	Y	-	Y	-	-	Y	-	-	-	Y	Y	Y	Y	Y	Y	Y
Clarithromycin	-	Y	-	Y	-	-	Y	-	-	-	Y	Y	Y	Y	Y	Y	Y
Azithromycin	-	Y	-	Y	-	-	Y	-	-	-	Y	Y	Y	Y	Y	Y	Y
Sum of macrolides	-	Y	-	Y	-	-	Y	-	-	-	Y	Y	Y	Y	Y	Y	Y
AMC indicators under surveillance	0%	83%	0%	100%	0%	0%	100%	0%	0%	0%	83%	100%	100%	100%	100%	100%	100%

PRIMARY CARE																	
**Antimicrobial resistance indicators**	**AT-3**	**HR-6**[(a)]	**CZ-7**	**DK-8**	**DE-10**	**GR-12**	**IT-14**	**LT-17**	**LT-19**	**PL-25**	**PT-26**	**SP-30**	**SP-31**	**SP-32**	**SP-33**	**SP-34**	**SP-36**
CPE	-	Y	-	Y	-	-	Y	-	-	-	-	-	Y	-	Y	-	Y
CIPR *Escherichia coli*	-	Y	-	Y	-	-	Y	-	-	-	-	-	Y	-	Y	Y	Y
ESBL *Escherichia coli*	-	Y	-	-	-	-	Y	-	-	-	-	-	Y	-	Y	Y	Y
ESBL *Klebsiella pneumoniae*	-	Y	-	-	-	-	Y	-	-	-	-	-	Y	-	Y	Y	Y
MRSA	-	Y	-	Y	-	-	Y	-	-	-	-	-	Y	-	Y	Y	Y
AMR indicators under surveillance	0%	100%	0%	60%	0%	0%	100%	0%	0%	0%	0%	0%	100%	0%	100%	80%	100%

Antimicrobial use was assessed as DID: DDD (defined daily doses) per 1000 inhabitants; Antimicrobial resistance incidence density was assessed as number of outpatients with positive clinical isolates per 1000 inhabitants; CPE: carbapenemase-producing Enterobacteriaceae; ESBL: Extended-spectrum beta-lactamase; CIPR: Ciprofloxacin-resistant; MRSA: meticillin-resistant *Staphylococcus aureus*; (a) Antimicrobial resistance data are provided for a sample population over 280,000 people; Y: data informed; (-): no data informed.

Piloting phase

Indicators

The key indicators will be provided on a quarterly basis, by means of providing both numerator and denominator for each indicator (no direct input for any indicator) in a Microsoft Excel spreadsheet on a first stage, while the online spreadsheet application is developed.

Once the Excel spreadsheet that accompanies this document is completed, each contributor will send it to the data coordinator within two months after each quarter:

Quarterly data	Data Submission
1st. January-February-March	April-May
2nd. April-May-June	July-August
3rd. July-August-September	October-November
4th. October-November-December	January-February

A. Indicators for Antibiotic Pressure (Consumption):

Definition

DDD/1000 stays (occupied bed days, OBD)* in the trimester, in hospital settings

DDD/1000 inhabitants in the trimester, in primary care settings.

*OBD: is the sum for the quarter of the daily figures for the number of beds occupied by patients. A bed temporarily used by a day case during the day but otherwise not in use by an inpatient is counted as unoccupied. So, wards like emergency services, observation etc. will be excluded.

Indicators for hospital settings

- Overall antibiotics consumption in hospitals (DDD/1000 stays per trimester):

**Overall DDD/1000 OBD of antibiotics [a]**	**Total DDD of antibiotics (J01) a trimester x 1000/No. of total stays during the trimester**

- Key antibiotics:

Piperacillin-tazobactam

Amoxycillin-clavulanate

Carbapenems (ertapenem + imipenem + meropenem + doripenem)

3rd and 4th cephalosporins (ceftriaxone + cefotaxime + ceftazidime + cefepime)

Quinolones (ciprofloxacin + levofloxacin + moxifloxacin)

Vancomycin

Colistin

**DDD/1000 OBD of a certain antimicrobial drug [a]**	**Consumption (grams) of a certain antimicrobial drug a trimester x 1000/DDD of this antimicrobial drug x No. of total stays during the trimester**

[a] The unit of measure will be the Defined Daily Doses (DDD), following the Anatomical Therapeutical Chemical Classification (ATC/DDD) methodology. Index 2018. WHO Collaborating Centre for Drug Statistics Methodology. Oslo; 2017 Available at: https://www.whocc.no/atc_ddd_index/

Indicators for primary care settings

- Overall antimicrobial drugs consumption in primary care (DDD/1000 inhabitants per trimester).

**Overall DID of antibiotics in primary care**	**Total DDD [a] of antibiotics (J01) x 1000 / No. of inhabitants in the healthcare area during the trimester**

- Key antibiotics:

Amoxycillin-clavulanate

Quinolones (ciprofloxacin + levofloxacin + moxifloxacin)

Macrolides (erythromycin + clarithromycin + azithromycin)

**DID of a certain antimicrobial drug**	**No. of packaging units sold of a certain antimicrobial drug x No. of pharmaceutical forms per package x grams of active ingredient in each pharmaceutical form x 1000/DDD [a] of this antimicrobial drug x No. of inhabitants in the healthcare area during the trimester**

[a] The unit of measure will be the Defined Daily Doses (DDD), following the Anatomical Therapeutical Chemical Classification (ATC/DDD) methodology. Index 2018. WHO Collaborating Centre for Drug Statistics Methodology. Oslo; 2017 Available at: https://www.whocc.no/atc_ddd_index/

B. Indicators Of Antimicrobial Resistance (AMR):

Definition

Number of isolates in clinical samples per patient/1000 stays (occupied bed days) in hospital.

Number of isolates in clinical samples per patient/1000 inhabitants in primary care.

AMR indicators in hospitals

Carbapenemase-Producing Enterobacteriaceae

ESBL *E. coli*

ESBL *K. pneumoniae*

Carbapenem-resistant *A. baumannii*

Carbapenem-resistant *P. aeruginosa*

Meticillin-resistant *S. aureus* (MRSA)

Vancomycin resistant enterococci (*E. faecalis* and *E. faecium*)

**Incidence density of inpatients with a certain resistant microorganism**	**No. of inpatients with a certain resistant microorganism infection/colonisation x 1000/No. of total stays during the trimester**

Duplicated bacterial strains in the same patient (isolation of the same pathogen, regardless of the collection site, with same susceptibility test results ≤365 days) will be not included in the surveillance, as well as those samples from environment and colonisation screening (nasal smears, pharyngeal swabs, pressure ulcers, perianal or rectal smears, tracheostomy, etc.) as these outcomes will depend on the surveillance intensity of each hospital.

AMR indicators in primary care

Carbapenemase-Producing Enterobacteriaceae

Ciprofloxacin-resistant *E. coli*

ESBL *E. coli*

ESBL *K. pneumoniae*

Meticillin-resistant *S. aureus* (MRSA)

**Incidence Density of outpatients with a certain resistant microorganism**	**No. of outpatients with a certain resistant microorganism infection/colonisation x 1000/No. of inhabitants**

Duplicated bacterial strains in the same patient (isolation of the same pathogen, regardless of the collection site, with same susceptibility test results ≤365 days) will be not included in the surveillance.

The number of inhabitants to be considered in each region/country will be the number of individual public healthcare cards or similar.

EU-JAMRAI

Joint Action on Antimicrobial Resistance and Healthcare-Associated Infections

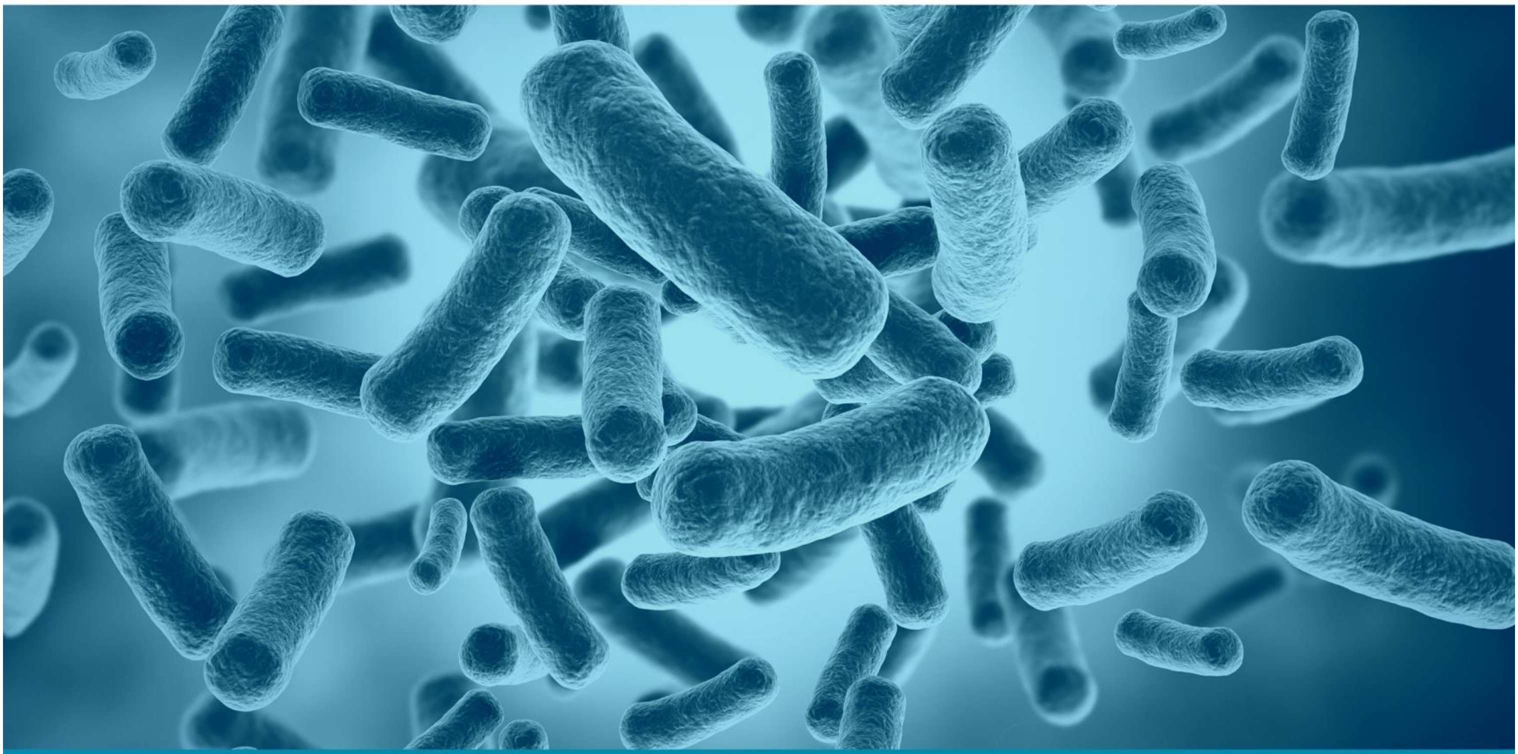

This document arises from the Joint Action on Antimicrobial Resistance and Healthcare-Associated Infections (EU-JAMRAI), which has received funding from the European Union, in the framework of the Health Program (2014-2020) under the Grant Agreement Nº761296. Sole responsibility lies with the author and the Consumers, Health, Agriculture and Food Executive Agency is not responsible for any use that may be made of in the information contained therein.

Co-Funded by the
Health Programme
of the European Union
